# Rupture Risk Assessment of Cervical Spinal Manipulations on Carotid Atherosclerotic Plaque by a 3D Fluid-Structure Interaction Model

**DOI:** 10.1155/2021/8239326

**Published:** 2021-01-02

**Authors:** Yili Chen, Shaoqun Zhang, Yang Chen, Yonghua Lao, Xuecheng Huang, Xiaoyu Huang, Qiming Liao, Yikai Li

**Affiliations:** ^1^Wang Jing Hospital of China Academy of Chinese Medical Sciences, Beijing 100102, China; ^2^Shenzhen Traditional Chinese Medicine Hospital, Shenzhen 518110, China; ^3^Beijing University of Chinese Medicine, Beijing 100102, China; ^4^Department of Biomedical Engineering, School of Material Science and Engineering, South China University of Technology, Guangzhou 510006, China; ^5^Department of Human Anatomy, Southern Medical University School of Basic Medical Sciences, Guangzhou 510515, China; ^6^1118, 11th Floor, Building 4, No. 156, Hanxing Middle Road, Zhongcun Street, Panyu District, , Guangzhou 510515, , China; ^7^School of Traditional Chinese Medicine, Southern Medical University, Guangzhou 510515, China

## Abstract

**Method:**

The FSI model, based on MRI data of an atherosclerosis patient, was used to simulate the deformations of the plaque and lumen during the process of two kinds of typical cSMT (the high-speed, low-amplitude spinal manipulation and the cervical rotatory manipulation). The biomechanical parameters were recorded, such as the highest wall shear stress (WSS), the maximum plaque wall stress (PWS), the wall tensile stress (Von mises stress, VWTS), and the strain.

**Result:**

The max_WSS was 33.77 kPa in the most extensive deformation. The highest WSS region on the plaque surface was also the highest PWS region. The max_PWS in a 12% stretch was 55.11 kPa, which was lower than the rupture threshold. The max_VWTS of the cap in 12% stretch which approached the fracture stress level was 116.75 kPa. Moreover, the vessel's max_VWTS values in 10% and 12% stretch were 554.21 and 855.19 kPa. They were higher than the fracture threshold, which might cause media fracture. Meanwhile, the 7% stretched strain was 0.29, closed to the smallest experimental green strains at rupture.

**Conclusion:**

The carotid arteries' higher stretch generated the higher stress level of the plaque. Cervical rotatory manipulation might cause plaque at a high risk of rupture in deformation after 12% stretch and more. Lower deformation of the plaque and artery caused by the high-speed, low-amplitude spinal manipulation might be safer.

## 1. Introduction

Cervical spinal manipulative therapy (cSMT), as an effective treatment for neck pain and headaches, has been widely accepted [1]. Historically, according to a case report [2], in a high-risk stroke patient, cSMT could cause serious diseases such as a transient ischemic attack, paralysis, and shock. One of the major concerns for the safety of cSMT is the risk of stroke [3]. Therefore, there is still a controversy about whether cSMT could lead to a stroke in carotid atherosclerosis. The rupture of plaque [4] caused most of the cerebral infarcti ons. However, the impacts of cSMT on carotid atherosclerotic plaque have not yet been clear cut. There is still safe hidden trouble between cSMT on carotid atherosclerotic plaque and rupture. Some therapists recommend that cSMT should not proceed in carotid atherosclerosis because of the risk of stroke. However, others suggest that normative therapies are safer in the asymptomatic patient with stable plaque.

Some researches [5, 6] had reported the force and strain of the high-speed, low-amplitude spinal manipulation on the vertebral artery. The vertebral artery (VA) strain and stretch were smaller than the ROM's (rotate the neck to the end range of motion). The strain was one-ninth of the value of mechanical failure. This normative cSMT preceded by the therapist would not disrupt the VA. Otherwise, the blood flow change in VA and internal carotid artery (ICA) following some cSMT has been studied [7–9]. However, there is still a lack of research about the impact of the biomechanical changes of the atherosclerosis plaque and the carotid artery (CA) lumen following cSMT.

In our study, the fluid-structure interaction (FSI) model in finite element analysis was used to assess the biomechanical changes. It could be used to analyze the biomechanics of carotid plaque in recent studies. There was a 3D multicomponent fluid-structure interaction (FSI) model that could assess plaque structural stress behaviors [10]. The maximum principal stress on the plaque surface was well defined as plaque wall stress (PWS). The PWS, as a critical stress situation, is related to the prior rupture. Furthermore, it is found by several groups that the higher PWS obtained from FSI models were linked to plaque rupture [11]. Meanwhile, high shear stress might lead to endothelial dysfunction, lumen surface weakening, and rupture [12]. Moreover, as a predictor of carotid plaque rupture, the PWS is better than the flow shear stress [13]. It had been found that the highest wall shear stress (WSS) location where the ulcer was generated exclusively made plaque to be more vulnerable [14]. Therefore, the WSS might become a potential parameter for predicting rupture. 300 kPa was utilized as the stress value of the plaque rupture threshold in many FEA studies [15]. The plaque in which the stress situation had been higher than 300 kPa was considered a high risk of plaque rupture [16]. Additionally, there was a cohort study focused on the fracture stresses/stretch properties of atherosclerotic plaques [17]. It has been hypothesized that the plaque wall region with the concentrated mechanical stress and exceeded material strength might be ruptured first. Local high-stress concentration was one of the most acknowledged biomechanical factors of plaque rupture. Therefore, it is essential to simulate the plaque's biomechanical stress by proceeding cSMT for predicting rupture sites. To date, the impacts of cSMT on the plaque stress have not been well characterized.

In this research, two typical cSMT are studied. One is the high-speed force and low amplitude of thrust [18], which has been researched [19] by Herzog. Another one is cervical rotatory manipulation, a useful and common Chinese cSMT [20], which would perform a torsion force in the ROM. The main difference is the mean value of the deformation of carotid arteries caused by two manipulations. The value of cervical rotatory manipulation is larger than the other because of the larger neck rotation range. According to the research [19], the mean value of ICA stretch during the high-speed, low-amplitude spine manipulation, and the ROM were 2% ± 4% and 7% ± 9%. It is significant to evaluate PWS, WSS, fracture stress values, and stretch of all plaque components under cSMT to investigate the impacts of the carotid plaque and the CA following cSMT. If these values were much higher than those rupture thresholds, the cSMT is not safe for atherosclerosis.

This study's novelty used a 3D FSI model to analyze the plaque's stress and stretch during cSMT. The atherosclerotic plaque model could be reconstructed to analyze the stress distribution to predict the rupture by accurately delineating advanced MRI images. Using the FSI model to simulate the cSMT is a new method to study the plaque's impact. Also, the FSI solutions were meaningful to do rupture risk assessments. This study is aimed at quantifying the plaque's biomechanical situation and changes by these two different kinds of cSMT.

## 2. Methods

### 2.1. Data Acquisition

An asymptomatic patient (age: 68 years, male) with a single typical carotid plaque on carotid bifurcation was recruited from Nanfang Hospital. There was no treatment or intervention for the patient. The study protocol was approved by the local Research Ethics Committee, and written informed consent was given. The Research Ethics number is NFEC-2018-159, and the Clinical Trial Registry number of this study protocol was ChiCTR1800016407.

A routine physical examination had been done during recruitment. It acquired the patient's basic vital signs, such as blood pressure (125/62 mmHg) and heart rates (80 beats per minute). All carotid arteries had been observed under color Doppler ultrasonography to measure the mean flow velocities in two positions (the neutral position and the ROM position), shown in Table 1. Using a protocol approved by the institutional review board with informed consent obtained at a location, the 3.0 T MRI data of the carotid atherosclerotic plaque was acquired at the Nanfang Hospital. The sequential slices with no gap of the common carotid artery (CCA), internal carotid artery (ICA), and external carotid artery (ECA) were acquired. Besides, time of flight (TOF) sequence-weighted (repetition time (TR)/effective echo time (TEeff)/echo train length (ETL):29 ms/4 ms/20 mm) was performed to identify the location of the carotid bifurcation and plaque. T1 weighted (TR/TEeff/ETL:800 ms/10 ms/20 mm) and T2 weighted (TR/TEeff/ETL: 4800 ms/60 ms/20 mm) with a voxel size of 0.3∗0.3∗2 mm used double-inversion recovery blood suppression. The 2D electrocardiogram- (ECG-) triggered, fat-suppressed fast spin-echo pulse sequences were employed. The parameters of the images were 512∗512 matrix size and 2 mm slice thickness. Moreover, we distinguished various plaque components such as fibrous cap, lipid core, calcification, and arterial wall according to the study by Gao [21].

### 2.2. 3D Plaque Geometry Reconstruction and Mesh Generation

**Figure 1:**
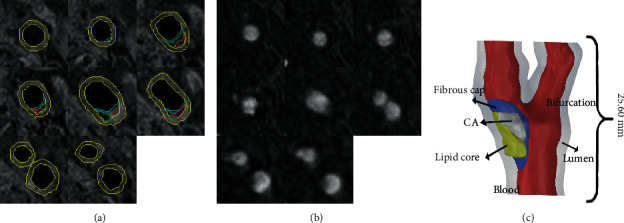
(a) T1WI segmentation of the whole plaque geometry, the artery, lipid core, fibrous cap, and calcification were colored in yellow, purple, blue, and green, respectively. (b) TOF segmentation of the plaque. (c) In the restructured model in Solidworks2017, the lipid core, fibrous cap (FC), calcification (Ca), and the blood were colored in yellow, blue, and white, respectively.

The reconstructive model of carotid plaque was based on the MRI data. According to the delineation of various plaque components [21], all image segmentation was marked into different masks in Mimics17.0, respectively, shown in Figure 1(a). The blood fluid, identified from the TOF images (Figure 1(b)), was used to construct. After all, components were identified and marked, the generated 3D elements of each component in Mimics17.0 were imported into Solidworks2017 for integration. As shown in Figure 1(c), a 3D reconstructed model was created. The most stenotic region was the carotid bifurcation caused by the plaque. Meanwhile, the lipid core, fibrous cap, and calcification were colored in yellow, blue, and white, respectively, in the reconstructive model. ANSYS14.5 generated the 3D surfaces, volumes, and computational mesh. The body sizing restrictions for the lipid core, fibrous cap, and calcification were set as 0.15 mm/0.18 mm/0.5 mm. With the algorithm of patch confirming, the structure model has meshed into 340,000 tetrahedrons and 500,000 nodes. The tetrahedrons and the nodes of lipid core, fibrous cap, and calcification were 65,000/95,000, 46,000/70,000, and 65,000/95,000, respectively. As the fluid domain, the blood was meshed in the size of 0.3 mm and the tetrahedrons method. It has meshed into 390,000 3D tetra cells and 71,000 nodes.

### 2.3. Material Properties and Boundary Conditions

**Figure 2:**
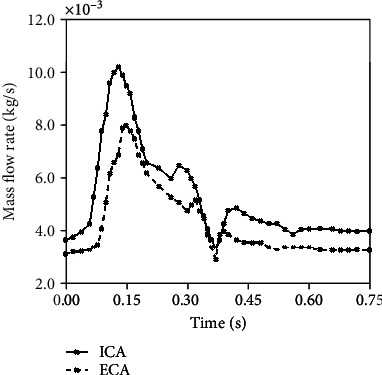
Simulation boundary condition for blood flow. The target mass flow rate for ICA and ECA for the model.

According to the research [22], the blood was considered an elastic, viscous, incompressible Newtonian fluid. The blood's viscosity was 4^∗^10^−3^ Pa.s and the density was 1,067 kg/m^−3^. The flow was treated as a transient and turbulent flow. Moreover, the outlet simulation was set as the time-dependent mass flow rates of ICA and ECA, which were the generalized waveforms reported by Lee [23], shown in Figure 2. The pressure conditions and the heart rate of the individual were imported. Hence, a total of 150-time steps (0.05 s per step) were used for a cardiac cycle. These boundary conditions ensured a more realistic simulation and a much easier convergence.

Based on the published experimental results [24], a 5-parameter Mooney-Rivlin model was used to describe the carotid arterial wall. The material property was assumed to be nonlinear, isotropic, and incompressible. The strain energy function for the model is given by

(1) $$\begin{array}{c} W=C_{10}\left(I_{1}-3\right)+C_{01}\left(I_{2}-3\right)+C_{20}{\left(I_{1}-3\right)}^{2}+C_{11}\left(I_{1}-3\right)\left(I_{2}-3\right)+C_{02}{\left(I_{2}-3\right)}^{2}+\frac{1}{d}{\left(J-1\right)}^{2}. \end{array}$$

The material constants were *C*_10_ = 50,445 Pa, *C*_01_ = 30,491 Pa, *C*_20_ = 40,000 Pa, *C*_11_ = 120,000 Pa, *C*_02_ = 10,000 Pa, and *d* = 1.44*e*^−7^. Each component of the plaque's material parameters was chosen to match the experimental measurement and published research [25]. The lipid core and fibrous cap were assumed to be soft. Young's modulus and Poisson ratios of lipid core and fibrous cap were 2 kPa/0.49 and 270 kPa/0.3, respectively. And the calcification was considered stiffer with 690 MPa Young's modulus and 0.3 Poisson ratio. The contacts between lipid core and calcification were set as frictionless. The other contacts were set as bonded.

### 2.4. FSI Simulation and Solution Method

In this study, to simulate cSMT, the deformation was set normal to ICA's flat surface and ECA's terminal points. And the fixed flat surface was set at the bottom of the model, the initial point of CCA. The stress and the strain in each value of stretch could be reported in this solution. The high-speed, low-amplitude spinal manipulation of the neck has been simulated that ICA's deformation during this cSMT was 2% ± 4%[19]. Another cSMT was cervical rotatory manipulation, in which simulated deformation was reported as the max stretch of ICA during the ROM (7% ± 9%). The 2% and 6% deformations of the carotid artery could be represented by the mean and maximum changes in the process of the high-speed, low-amplitude spinal manipulation of the neck. The 7% and 16% deformations could also be represented as a simulation of the ROM and the Chinese SMT (rotate the neck in the cervical spine's ROM without a torsion force). The advantage of these simulated deformations of carotid arteries was closer to reality. There were few studies within human about the characteristics of the deformation of VA and CA while proceeding cSMT except stretch. Therefore, vertical deformations were in the simulation process. The PWS, WSS, fracture stresses, and stretch of the plaque corresponding to these deformations were recorded. The PWS, the maximum plaque stress, was obtained from the fibrous cap covering lipid core and calcification. The WSS obtained from the FSI solution was defined as the max flow shear stress on the surface of the fibrous cap. Fracture stresses were defined as wall tensile stress in the von mises stress form, named VWTS [22]. It could represent the plaque interior stress distribution. During the process, the plaque's maximum VWTS in a cycle was chosen to associate with the interior rupture. Stretch and strain were reported to compare with some ultimate tensile experiments of carotid plaque mechanics properties.

## 3. Result

All models had been converged. The FSI model's flow velocities were similar to the velocities measured by color Doppler ultrasonography in both positions, shown in Table 1. And the mesh density sensitivity analysis had been done in the FSI model. All stress values calculated from three different mesh sizes (0.25/0.3/0.5 mm) in the same region were less than 5% different. The model in this study would be considered verified.

**Figure 3:**
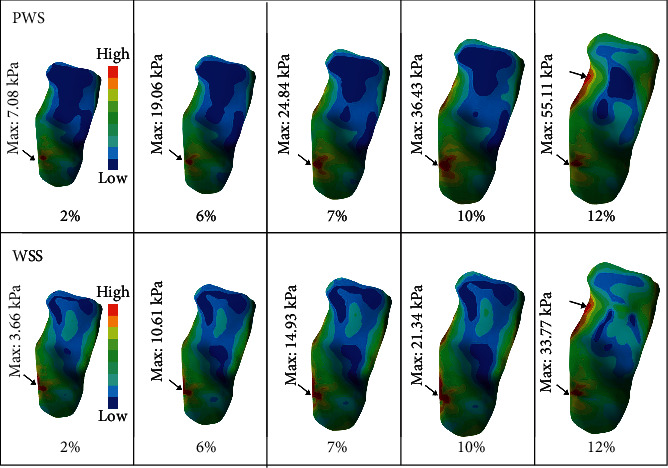
The PWS and WSS distributions in each stretch simulated by the FSI models. The maximum region is indicated by arrows. The values of stress were heightening with larger stretch. The first row shows the PWS on the plague surface; the second row shows the WSS distribution during stretching.

**Figure 4:**
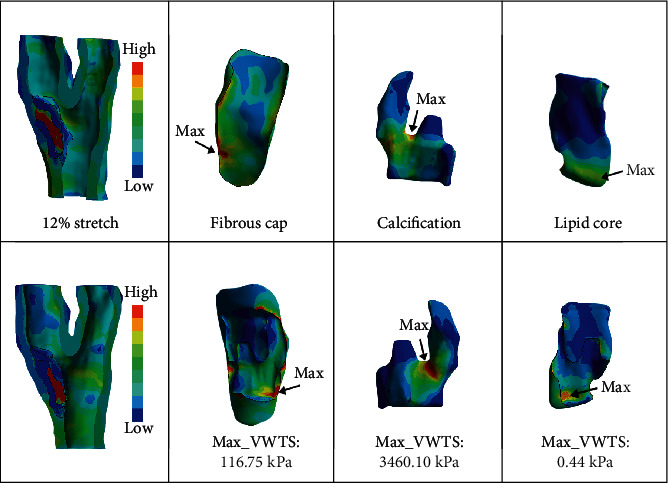
The VWTS distribution in a 12% stretch was calculated by the FSI models. The maximum region is indicated by arrows. All components of the model are shown their front and back in one column. The first column shows the model's whole geometry after stretching; the second column is the local stress of the fibrous cap; the third column is about the calcification; the fourth column shows the local stress on the lipid.

Some elements could not be stretched too much due to some components' irregular shape and this model's high precision. The FSI model had been stretched at most in the total deformation of 12%. The plaque situation in the process of 2% to 12% of deformation had been all recorded. Deformations of 2%, 6%, 7%, and 12% were chosen to study. 2% and 6% were represented by high-speed, low-amplitude spinal manipulation. 7% and 12% were represented in the other. Obtained from the FSI model, the WSS and the PWS of each stretch were shown in Figure 3. The highest PWS was concentrated in similar regions where the thinnest fibrous cap was with a large lipid core and calcification. With the highest PWS indicated by the arrow, the fibrous cap surface would be considered the easiest rupture site of the plaque. The max_VWTS of each component and the strain of the fibrous cap were recorded in Table 2. The maximum value of VWTS for a 12% stretch in this solution was presented in Figure 4. The VWTS was the highest at the calcification and the lowest in the lipid core. Otherwise, the max_VWTS and strain of the vessel of each stretch were recorded in Table 2.

## 4. Discussion

Based on the stress analysis, the WSS was concentrated in the most narrowing region, the upstream of the plaque. The WSS distributions of plaque were calculated in different stretches. The max value of WSS was 33.77 kPa in the most extensive deformation. In the study [26], a significant shear stress value of the high-risk rupture was higher than 1.12*e*+02 dnyes/cm^2^ calculated by CFD. If WSS in the shoulder of the plaque was over the threshold, it might cause endothelial damage. Nevertheless, there is still no consensus within humans studied on the range of plaque shear stress. The max_PWS of each stretch was also calculated. As shown in Figure 4, the highest WSS region on the plaque surface was also the region with the highest PWS. It would be the most likely happened endothelial damage, even ruptured. In this region, the thinnest fibrous caps were contained the thickest calcification and lipid core. The more massive stretch inputted, the higher PWS and WSS were generated. The model's max_PWS was 55.11 kPa in the 12% stretch, as it simulates a rotating neck to the end range of the motion. Also, after 10% stretch inputted, the stress concentration region had been changed. There is more stress concentrated on the upside of the shoulder. Currently, the most used threshold value within FE studies is 300 kPa (0.3 MPa) proposed by Cheng [27]. Another study suggested that 300 kPa could be regarded as a stress level for instability of plaque rather than rupture threshold [28]. Furthermore, Deepa [16] argued that the plaque with a stress value of 300 kPa is at a high risk of plaque rupture. It could be chosen as a trigger of initial plaque rupture. Meanwhile, Vengrenyuk [29] predicts that the fibrous cap with calcification suffered apoptosis in the high-stress level close to 600 kPa. According to these thresholds, the max_PWS in the 12% stretch was much more lower than 300 kPa. Even that this plaque was stable contained with a large calcification. Therefore, in this model, rotating the neck to the end range of the motion would increase the PWS and WSS values on the plaque surface, but it would not cause rupture or affect plaque stability.

On the other hand, the arteries and plaque's fracture stress/stretch properties are significant for predicting plaque fracture. Plaque fracture would lead to ulcer, hemorrhage, and affect plaque stability, even rupture. The arteries and plaque's ultimate stress/strain could be recognized as a trigger for the rupture. The max_VWTS, the max interior stress of each tissue, could be used to assess the rupture for atherosclerosis. The equivalent elastic strain of the tissue would be calculated and associated with the ultimate stretch test in the axial direction. The experimental green strains at the rupture of fresh carotid artery plaques varied from 0.299 to 0.588, and the Cauchy stress observed was between 131 and 799 kPa [30]. The lowest fracture of the fibrous cap (254.80 ± 79.80 kPa at strain 1.18 ± 0.10), but the stress and stretch fracture values at the joint calcification and fibrous cap are the smallest (179.00 ± 56.00 kPa and 1.02 ± 0.005) [15]. Only the max_VWTS of the cap in 12% stretch was approached the fracture stress level, shown in Figure 4. The stress distribution showed the stress concentration was in the cap's back, where the thin fibrous cap and the thickest calcification and lipid core were joined. This region was at a high risk of fracture. Meanwhile, the 7% stretched strain was 0.29, equal to the smallest experimental Green strains at rupture. It might be closed to the ultimate strain level. With the larger stretch inputted, this region will be fractured earliest. Rotating the neck in the end range of motion might lead to this plaque fracture, even though a torsion force needs to be applied.

According to the research by Teng [12], the fracture stresses were a mean of 1996 ± 867 kPa for the vessel's adventitia. And the mean values of 519 ± 270 kPa and 1230 ± 533 kPa for the media. The fracture strain was 1.50 ± 0.22. All results of the simulation were recorded in Table 2. The larger the stretch inputted in the model, the larger the stresses and strains of all components. The stresses and strains of all components calculated under 10% stretch were not higher than any thresholds mentioned above, except the max_VWTS of Ca and vessel. In this study, the vessel, considered a hyperelastic lumen, has not been divided into the media and the adventitia. The max_VWTS values of the vessel in 10% and 12% stretch were 554.21 and 855.19 kPa. The 10% stretched vessel's stress was approached the mean value of the media fracture threshold (519 ± 270 kPa). And the value of a 12% stretched vessel was higher than the max value of the media fracture. It might occur a medium fracture in this stretch, which means rotating the neck to the end range might injure the carotid arteries' media. And the fibrous cap's max_VWTS in 12% stretch was lower than the Cauchy stress, but close to the fracture value. Therefore, the shoulder of the plaque was the most likely injured region by the rotation. It was defined as the joint of the fibrous cap and the lumen, shown in Figure 4. Otherwise, the max values generated in calcification were much higher than the others. It could be associated with its material property, which was stiffer than other components. The strain of calcification was too low. The max strains of lipids were higher than others because of their soothing properties. However, there was still a lack of researches on the ultimate stress/strain of the calcification and lipid core in human carotid plaques. These two issues could not be evaluated whether they are at a high risk of rupture level or on the threshold of fracture in the simulation. There was no result of the plaque higher than any threshold in this solution. We thought the stress/strain of this model would not result in a tensile failure. Meanwhile, the symptomatic plaque with the high-stress concentration was easier to rupture than the asymptomatic plaque. Gao [31] found local maximum stress values predicted in the fibrous cap region that the stress in symptomatic patients was higher than the asymptomatic patients (200 ± 43 kPa vs. 127 ± 37 kPa). Though there was no threshold of the symptomatic plaque, the max_VWTS of the fibrous cap calculated in 12% stretch was lower than 200 ± 43 kPa. This stretch would not cause the asymptomatic plaque to become symptomatic, one in the local stress level.

Plaque rupture is a complex process. Many factors, such as fibrous cap thickness and lipid core volume, would influence the plaque. Plaque stable structure is also an essential factor influencing the rupture risk. Simple stress analysis could not directly demonstrate that simply stretching of the carotid arteries by cSMT will cause the plaque to rupture. However, no matter which kind of cSMT, the larger deformations of arteries inputted, the more enormous impacts will be applied to the plaque and lumen. The plaque will be at a higher risk of rupture and fracture. In this study, the stretch of two different cSMT was only simulated without a subsequent torque. The cervical rotatory manipulation would rotate the neck to the end range of motion and apply a torque force, which might cause the plaque and lumen at a higher risk of rupture and fracture. A subsequent torque might lead the plaque situation to be further worse. Based on the stress-strain values calculated in the models, 2% to 6% stretch would not cause the plaque a high risk of rupture and fracture, but 12% stretch might. Therefore, the high-speed, low-amplitude spinal manipulation of the neck might be relatively safer than the cervical rotatory manipulation because of the smaller deformation of the carotid arteries.

The study's major limitations are as follows. (1) It might need to have post-cSMT clinical data to support modeling results. Clinical treatment under the rupture risk of the plaque was not allowed. (2) The max stretch of ROM is 16%. The 16% stretch in this model could not be convergence based on the high precise model due to too small elements and too many nodes that could not be deformed much more. (3) 300 kPa can only be served as a reference rupture stress value; more critical thresholds should be provided for rupture assessment. (4) More reconstructive samples were needed to do a statistical analysis for the safety assessment of cSMT.

## 5. Conclusion

In conclusion, based on in vivo multisequence MRI images, the FSI model of this patient could evaluate the stress distribution and strain values precisely during the process of cSMT. For this case, PWS, WSS, and VWTS of each component in different stretches on two cSMT were acquired. While only in a 12% stretch, the plaque was at risk of rupture. The larger stretch would be possibly dangerous for the plaque. According to the models' stress analysis, the high-speed, low-amplitude spinal manipulation of the neck would be relatively safer for this patient; the cervical rotatory manipulation or rotating the neck to exceed the end range of motion might make the plaque at a rupture risk. Before proceeding with cSMT, FEA is a useful screening method for safety assessment for atherosclerosis.

## Figures and Tables

**Table 1:** Velocity of the carotid arteries in model and measured by ultrasonography between two different position.

Position	The neutral position		The end range of motion	
cm/s	The model	Ultrasonography	The model	Ultrasonography
V_mean (CCA)	93.65	90.60 ± 11.50	60.20	60.00 ± 7.20
V_mean (ICA)	76.63	73.44 ± 5.60	47.34	48.50 ± 6.60
V_mean (ECA)	83.24	80.80 ± 8.20	48.57	50.00 ± 2.65

**Table 2:** Max_VWTS distributions and the strains of different stretches.

R of stretch	2%	6%	7%	10%	12%
VWTS_FC (kPa)	14.14	39.25	59.90	90.37	116.75
VWTS_Ca	317.14	1015.10	1543.80	2514.40	3460.10
VWTS_lipid	0.12	0.35	0.60	1.02	1.93
VWTS_Vessel	37.68	159.96	282.66	554.21	855.19
Strain_FC (mm/mm)	5.25*e*-02	0.15	0.29	0.35	0.44
Strain_Ca	6.42*e*-04	2.00*e*-03	3.01*e*-03	4.938*e*-03	6.825*e*-03
Strain_lipid	6.86*e*-02	0.18	0.30	0.51	0.97
Strain_Vessel	9.10*e*-02	0.23	0.30	0.39	0.47

## Data Availability

The data used to support the findings of this study are included within the article. If you need more other data, please contact us; they are partially available from the first and corresponding author upon request.
